# Dental Medicaments with Special Reference to Antiseptics

**Published:** 1891-04

**Authors:** J. W. Jungman

**Affiliations:** Cleveland, O.


ARTICLE IV.

### DENTAL MEDICAMENTS WITH SPECIAL REFERENCE TO ANTISEPTICS.

BY J. W. JUNGMAN, D. D. S., CLEVELAND, O.

Experiments of eminent bacteriologists and other scientists have proven, beyond a doubt, that bacteria cause and develop abscess of the alveolus, antrum, necrosis and caries of the teeth; therefore the dental surgeon should be familiar with this subject, should know the difference between antiseptics, escharotics, astringents, etc.

Antiseptics are medicinal agents capable of arresting fermentation, thereby preventing the decomposition of organic substances. When these agents are brought in contact with the disease germs they destroy their vitality. Included in this class are carbolic acid, creosote, salicylic acid, boric acid, iodol, peroxide of hydrogen, bichloride of mercury, and many others, but the above named being of more interest to the dentist.

In my practice, which has been of short duration, I have had several cases where antiseptics, astringents, escharotics, etc., were indicated, the principal case that of an abscess of the antrum. A gentleman about 62 years of age came to me with a fistulous opening in the region of the right superior molar which had fallen out by disease (pyorrhœa alveolaris). The bony cavity had nearly closed with the exception of a small crack large enough to admit a spatula. I pronounced it abscess of the antrum, and then proceeded to treat. The first thing I made the opening as large as the end of my little finger to afford free drainage; about half a drachm of pus exuded therefrom. I syringed the cavity thoroughly with a warm solution of $HgCl_2$, 1 to 3,000, then placed a bit of loose cotton in the cavity. This treatment was continued about a week, but not with the best of success, so I used an escharotic to break down that secretory membrane; for an antiseptic wash I used resorcin with good results.

This antiseptic is a chemical compound of phenol and aromatic series to which carbolic acid belongs. It is obtained from certain resins by action of fusing alkalies, and is in the form of tabular prismatic, shining crystals, somewhat sweetish to the taste and very soluble in water. This remedy is considered preferable to carbolic acid; it is very valuable to the dentist; it may be applied in all cases where antiseptics are indicated.

Carbolic acid is obtained from coal tar by fractional distillation and subsequent purification, being extracted from the heavy coal tar oils. When pure it is in the form of a colorless acicular interlacing crystals, which at 95° F.

become an oily liquid, possessing a strong odor and taste closely resembling creosote in characteristics and properties. although it is a different substance. Much of what is called creosote is nothing but impure carbolic acid. It is one of the most universal antiseptics now in use; it is not only an antiseptic, but an escharotic, styptic, stimulant, etc., therefore making it a very valuable remedy in dental therapeutics. When applied to carious dentures it not only obtunds sensibility, but arrests putrefactive changes in the devitalized structures and coagulates the albuminous elements at the ends of the dentinal tubuli. It relieves odontalgia when applied to the surface of an exposed and painful pulp; when applied to an exposed pulp it forms by escharotic action an eschar, which some regard as conductive to the recovery of the organ, while others regard the quiescent state it produces as an indication of the degeneration of the pulp, and hence prefer to use it in a diluted form. Many object to the odor of the acid, but oil of cloves added to it will disguise its taste and odor to some extent.

Phenol sodique is also extensively employed in dental practice. It is composed of pure melted carbolic acid, 5 parts; solution of caustic potash, 1 part; distilled water, 5 parts. We have many mouth washes, lotions, etc., which contain carbolic acid. The following wash can be used to good advantage:

| | | |
|---|---|---|
| R | Acid carbolic - - - - | gtt x |
| | Glycerine - - - - - | ʒ ij |
| | Aqua - - - - - - | ℥ v |

M. Sig. Use as a gargle.

The Robinson remedy, which contains equal parts of caustic potash and carbolic acid, has been proven to be a valuable remedy in the treatment of pyorrhœa alveolaris.

Creosote, like carbolic acid, is a valuable agent in dental therapeutics, although the use of the latter has in some respects superseded that of the former, and being very similar in their action with some advantage in the case of carbolic acid.

Salicylic acid is also a very valuable agent in dental surgery. It is obtained by combining carbolic acid and caustic potash and snbjecting this compound to dry carbonic acid, under the influence of heat and by a process of distillation which liberates the salicylic acid in crystals; the crystals are then washed, dissolved in hot water and by crystallization we have the light brown powder, which is then bleached until it is quite white, but most of that sold is of a light cream color with a reddish tinge. It has no odor, a slight taste, and is very soluble in water.

Eucalyptus is another popular agent. It is obtained from eucalyptus globulous found originally in Australia, known as the "blue gum tree." These trees are growing in the southern parts of Europe and the United States, and in Northern Africa their presence is said to be a prevention of malaria. The oil of eucalyptus, either alone or combined with iodoform, forms one of the most effective antiseptics in use for the treatment of putrescent pulps of teeth, alveolar abscess, pyorrhœa alveolaris, foul ulcers, etc. Iodoform is a preparation of iodine, being obtained by action of chlorinated lime upon an alcoholic solution of iodide of potassium, heated at 104° F. It is in the form of small crystals of a yellow color, with an unpleasant taste and soft to the touch. It is volatile and soluble in alcohol, chloroform and ether, the fixed and volatile oils, but insoluble in water. This antiseptic possesses no escharotic properties sufficient to cause any irritation or destruction of the soft parts. Dr. C. N. Peirce recommends iodoform ground up with equal parts of oil of cloves and oil of eucalyptus, which forms a substance of a soft, cheesy consistency, a portion of which can be introduced to an inflamed part on a point of a small broach. Iodoform is also used as an anodyne for the relief of pain following the extraction of teeth affected with periodontitis. A great objection to iodoform is the unpleasant odor, but this can be disguised to some extent by adding a drop of oil of rose, oil of lavender, oil of cinnamon, or finely ground coffee.

Peroxide of hydrogen is also a valuable agent and at present very extensively used, although it was discovered in 1818. It is obtained by rubbing up peroxide of barium with distilled water so as to form a liquid paste, which is added gradually with constant stirring to distilled water. Acidulated with one-third of its weight of hydrochloric acid, contained in a vessel immersed in a freezing mixture when the muriatic acid is saturated, a fresh quantity of the acid in concentrated state is added, then more of the peroxide of barium, and then the operation repeated till the solution will hold no more chloride of barium, which is deposited by a mixture of ice and salt, except a small portion which is gotten rid of by adding sulphate of silver; the filtered liquid is then concentrated by sulphuric acid, and then the water rising in vapor is absorbed and peroxide of hydrogen is obtained nearly pure in the form of a colorless liquid. Peroxide of hydrogen is used especially in the treatment of alveolar abscess, alveolar pyorrhœa, ulcerations of the mucous membrane, gangrene, cancrum oris, fungus growths, bleaching teeth, etc. The essential oils are valuable dental antiseptics, prinaipally the following: Cajeput, cassia, cinnamon, cloves, eugenol, eucalyptus, phenol, mustard, caraway, peppermint, sassafras, thyme, pennyroyal, turpentine and wintergreen. Oil of cassia, diluted with bland oil, like that of wintegreen, is a valuable dressing for putrid root canals and abscesses. Dr. Black recommends the following mixture:

| | | |
|---|---|---|
| R | Carbolic acid - - - | 1 part |
| | Oil cassia - - - - | 2 parts. |
| | Oil wintergreen - - - | 3 parts. |

Mix the oils and add the melted crystals of carbolic acid; this is known as the 1, 2, 3 mixture. Scientists have discovered several good antiseptics within the last two years, but will not dwell upon them this evening.—*Ohio Journal of Dental Science.*

